# A Systematic Approach of Data Collection and Analysis in Medical Imaging Research

**DOI:** 10.31557/APJCP.2021.22.2.537

**Published:** 2021-02

**Authors:** Manjunath K N, Chitra Rajaram, Govardhan Hegde, Anjali Kulkarni, Rajendra Kurady, Manuel K

**Affiliations:** 1 *Department of Computer Science and Engineering, Manipal Institute of Technology, Manipal Academy of Higher Education, Manipal, 576104, India. *; 2 *Department of Information Science and Engineering, NIE Institute of Technology, Mysuru, 570008, India. *; 3 *Consultant, AI in Radiation Oncology, Bengaluru, India.*; 4 *RTWO Healthcare Solutions, J P Nagara, Bengaluru,560078, India, 560086. *; 5 *Software Consultant, Bengaluru, India 560100. *

**Keywords:** CT Colonography, oral contrast, secondary dataset, 3D volume, the volume of interest

## Abstract

**Background:**

Obtaining the right image dataset for the medical image research systematically is a tedious task. Anatomy segmentation is the key step before extracting the radiomic features from these images.

**Objective:**

The purpose of the study was to segment the 3D colon from CT images and to measure the smaller polyps using image processing techniques. This require huge number of samples for statistical analysis. Our objective was to systematically classify and arrange the dataset based on the parameters of interest so that the empirical testing becomes easier in medical image research.

**Materials and Methods:**

This paper discusses a systematic approach of data collection and analysis before using it for empirical testing. In this research the image were considered from National Cancer Institute (NCI). TCIA from NCI has a vast collection of diagnostic quality images for the research community. These datasets were classified before empirical testing of the research objectives. The images in the TCIA collection were acquired as per the standard protocol defined by the American College of Radiology. Patients in the age group of 50-80 years were involved in various clinical trials (multicenter). The dataset collection has more than 10 billion of DICOM images of various anatomies. In this study, the number of samples considered for empirical testing was 300 (n) acquired from both supine and prone positions. The datasets were classified based on the parameters of interest. The classified dataset makes the dataset selection easier during empirical testing. The images were validated for the data completeness as per the DICOM standard of the 2020b version. A case study of CT Colonography dataset is discussed.

**Conclusion:**

With this systematic approach of data collection and classification, analysis will be become more easier during empirical testing.

## Introduction

**Figure 1:**
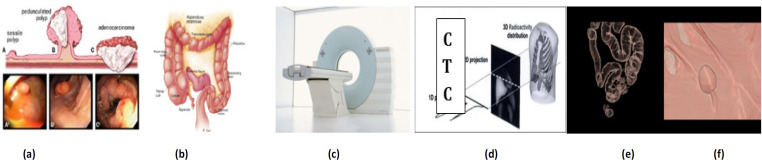
The CT Colonography Workflow. a) Polyp and colon cancer growth on the surface of the colon (source: Siegal, 2013 2), b) Colon preparation as per the ACRIN 6664 protocol, c) CT image acquisition, d) 2D image reconstruction from the projection data, e) the Desired volume of interest extraction from the 3D volume and f) Endoluminal view showing the polyp

Polyps are the precursors of cancer that happens in the colon (Figure 1a). Four parameters that decide the importance of a polyp are Size, shape, type, and grade of dysplasia. A polyp is diagnosed by conventional colonoscopy, or the non-invasive medical imaging-based technology called Computed Tomography Colonography (CTC), or virtual colonoscopy (Figure 1). The radiologists extensively use CTC images for the colon polyp analysis. By using image processing methods, the polyps are identified through automated software (CADe) with the help of a radiologist. CTC is not a diagnosis tool (CADx) that decides the stage of cancer. The steps in the CTC workflow includes the colon preparation (Fig. 1b) as per the standard CTC protocol (ACR, 2020), abdominal CT scan (Figure 1c) with approximately 6-7mSv (Milli Sievert) of radiation exposure to the patient, creating the 2D slices from the projection data (Figure 1d) and 3D volume reconstruction. Then the colon is segmented by sparing the non-volumes of interest (Figure 1e), visualized in 2D, and 3D to assess the polyps (Figure 1f). From the polyp parameters list, only the size and the shape are measured on CTC images. The rationale for the research was to provide improved image processing techniques to segment the colon and to identify the polyps accurately w.r.t shape and size (Siegel, 2020).

A systematic literature review on polyp analysis and the available dataset includes publications from MeSH (Medical Subject Headings), MEDLINE, PUBMED, SCIE, and EMBASE databases. Segmenting the volume of interest (VOI) from the set of images is an essential step before polyp analysis. The existing methods for colon segmentation are, K - Means clustering (Terry, 2004), Level set method (Franaszek et al., 2006), active contours and template matching (Chen et al., 2009; Breier et al., 2016), Fuzzy C thresholding (Franaszek et al., 2006), threshold-based region growing methods (Gross et al., 2009; Yoshida et al., 2012), volume thresholding and gradient-based edge detection methods (Cai et al., 2013) and Principal curvature (Lee et al., 2011). The effective colon segmentation at the mucous membrane still needs improved methods, because the base of the soft tissue structure is the key information to start measuring the polyp’s height and width (Lefere and Gryspeerdt, 2011). The dataset created in colon cancer screening has been archived by NCI (Smith et al., 2016; Johnson et al., 2008; Clark et al., 2013). There are other databases like BMIAXNAT (XNAT, 2020) and CERN’s Zenodo (CERN, 2020) repository.

The research objectives are colon segmentation, electronic cleansing of the tagged fecal matter, and measurement of the smaller polyps. Our objective was to systematically classify and arrange the dataset based on the parameters of interest so that the empirical testing becomes easier in medical image research. The required images were downloaded from the National Cancer Institute website’s TCIA CT Colonography collection (NCI, 2020). This source is a vast collection with more than eight hundred patients scanned in a mass colon cancer screening (with ACRIN 6664 protocol), and the ground truths are also available. The organization of this paper has two different sections. The first section discusses the details about the CTC datasets, the dataset selection methods, the curation of data, and validation against DICOM standards and the second section on the colon segmentation on different abdomen CT cases, along with the results.

## Materials and Methods

*A. Acquisition and Validation Methods*

CT Colonography data collection is made available from the Walter Reed Army Medical Center (WRAMC, Bethesda) in collaboration with NCI and NIH (courtesy: Dr. Richard Choi). It is a multicenter, clinical trial, anonymized images which were part of colon cancer screening done at WRAMC, and Naval Medical Center, San Diego, USA. The American College of Radiology and Imaging in Network (ACRIN) and the American College of Radiology (ACR) have jointly defined the protocol ACRIN 6664 (ACR, 2020; Johnson, 2016) for performing the CTC procedure. The protocol details are available in the article PMC2654614. The study included both symptomatic and asymptomatic male and female patients in the age group of 50 - 80 years. The study excludes patients with symptoms of the disease of the lower gastrointestinal tract and anemia, inflammatory bowel disease, familial polyposis syndrome, and prior colonoscopy in the previous five years cases. Scanning involved administering the patient with positive oral contrast for fecal tagging with Barium with medium-dose and full dose colon preparation, breath-hold technique to avoid the bowel peristalsis, and insufflating the air for colon distention. Then, the abdomen was CT scanned from the diaphragm to the pelvic region 12. Table 1 shows a summary of the image acquisition parameters from all the downloaded dataset.

The CTC scanning parameters were, ST=1-3mm, reconstruction interval of 1-1.25mm,mA = 200-300, effective mAs=50, kVp=120 and CTDIvol within 2mGy (Milli gray). The position of the patient scan includes Feet First Prone (FFP), Feet First Supine (FFS), Head First Prone (HFP), and Head First Supine (HFS) positions. The radiation exposure to the patient is approximately 6-7mSv (Milli Sievert). NCI has collected these datasets, and the data completeness is assured (Clark et al., 2013).

*B. Data Format and Usage Notes*

The diagnostic quality CTC images required for the research are downloaded from National Cancer Institute (NCI, 2020). All images in the dataset are in DICOM format with .ima and .dcm as the file extension. From the website, the user has to select the patient id from the available list, then a manifest file with .tcia extension gets downloaded. This file opens in the NBIA data retriever tool, which is a java based application. The organization of the images in the dataset follows the sequence Patient->Study->Series-CTImage. The metadata sheets are available, which contains the abstracts of radiology and optical colonoscopy reports describing the polyp occurrence (various sizes) in various segments of the colon. The confidence level of the radiologists who evaluated the polyp during colon cancer screening was least-certain, intermediate, and most certain.

*C. Data selection*

The ideal number of samples is essential in empirical testing. Statisticians calculated (n=150) the required number of samples for the research objectives. Systemic bias and sampling error are usually reported problems in inappropriate sample design. This bias is a problem while working with retrospect data. Even though there was option to select the dataset with only polyps, the bias is avoided by selecting cases without the polyp also. The sampling error is kept relatively small by selecting more number of samples (n=180). Datasets are carefully selected based on the diagnostic quality of the image, optimal colonic distention, ST, kVp, and pixel size, etc. There are more than 10,000 subjects (population) of different anatomical sites available. Further, the search was focused only on the CTC dataset, which resulted in the sample unit. The selection of datasets includes stratified sampling, in which the entire sample unit is divided into two homogeneous groups. Strata1 comprises of patients with polyps and colon cancer, and strata2 without these two. From the population, six hundred samples (N=600) were collected, out of which 540 patients were with polyps and 60 without polyps. The required sample size was 150. with N=600, we got N1=540, N2=60 (after Eq. 1). Thus the required sample sizes for strata1 and strata2 are 135 and 15, respectively, which is proportional to the size of the strata viz. 600:540.

$\mathrm{n1}=\left(\frac{\mathrm{N1}}{N}\right)\mathrm{*n}=\frac{540}{600}\mathrm{*150}=135$

and

$\mathrm{n2}=\left(\frac{\mathrm{N2}}{N}\right)\mathrm{*n}=\frac{60}{600}\mathrm{*150}=15$

*D. Data collection*

Datasets were collected for nearly five months through the questionnaire method. The key contact at NCI (help^@^cancerimagingarchive.net) clarified the doubts over the email. Search criteria (Table 2) include CT as an imaging modality, scans with and without oral contrast administration, ST of 1-3mm, and availability of dataset during 2002 – 2019. By looking into the CTC protocol followed (Johnson, 2016; Cash, 2010) and the data completeness, the images are carefully selected. Three hundred datasets are downloaded based on the calculated sample size and the source of the polyp as colon (by discarding the source as the rectum). As a first step, the authenticity (the reliability - who, how, and when data was collected, suitability - less noise and good tissue contrast, and adequacy - completeness and compatibility of DICOM images) of the data is checked.

*E. Data analysis and processing*

*E.1. Data analysis*

**Figure 2:**
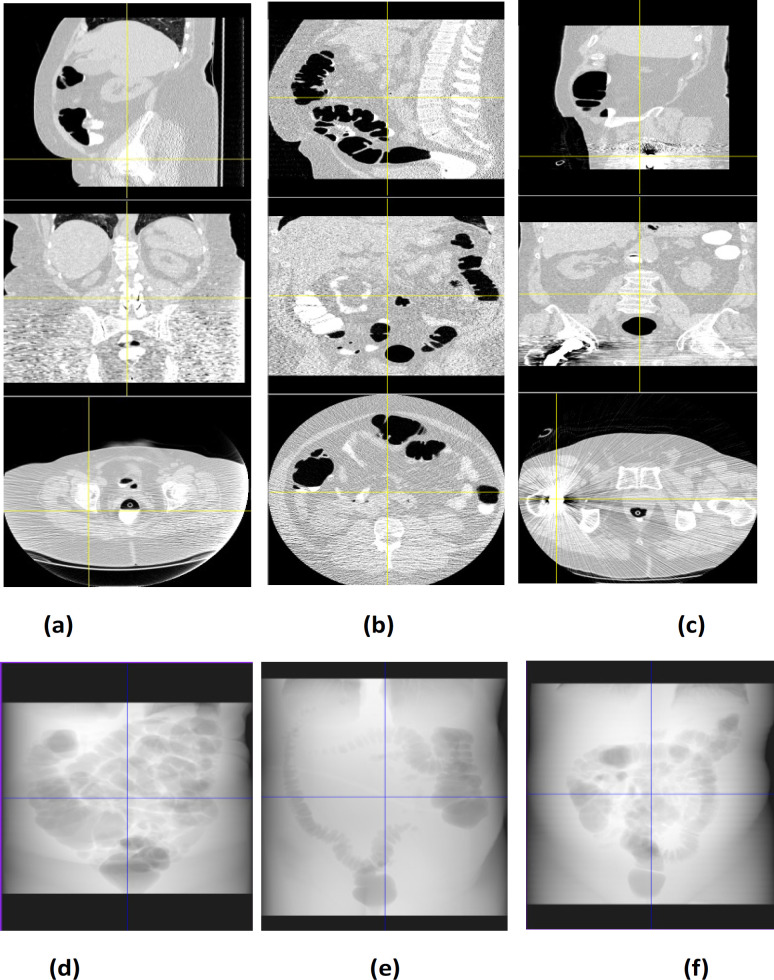
Bad Diagnostic Quality Images in Different Subjects (MPR and DRR images), a) Incomplete air insufflation, b) Patient too large and outside the scan field of view, c) Streak artifact, d) Incomplete distension of ascending and transverse colonic segments, e, f) Non-distended ascending colon

**Figure 3:**
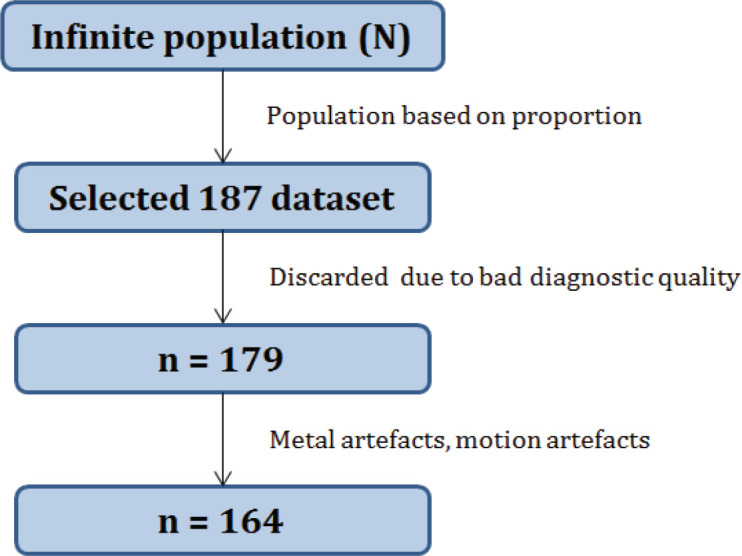
CTC Dataset Samples (n) Derived from the Population (N) after Discarding Non-Diagnostic Quality Images

CTC samples are manually checked for the diagnostic quality (possible artifacts, as mentioned in Table 3). As the diagnostic quality is not up to the mark, eight out of 187 cases are discarded. It is difficult to process such images. With this, the samples are reduced to 179. Fifteen datasets are rejected due to metal artifacts (Figure 2c), motion artifact, and quantum noise (Figure 2a). With this, the dataset count reduced to 164 (Figure 3). Editing any of the DICOM files either for the header details or the pixel details are not encountered. Also, it is unethical to modify the dataset. The images are contrast corrected for underexposed and overexposed regions (these regions resulted during CT image acquisition) without losing the soft tissue structure details on CT images. Gamma correction is applied to convert the stored pixel values in DICOM to the native display system. Without this, the same image looks different in different display systems (Kagadis et al., 2013) which may lead to wrong interpretation. A prototype software has been developed that has the basic features of medical image processing applications (Manjunath et al., 2017). In addition to testing the images in the prototype software, the images were checked syngoFastView from SIEMENS (Siemens, 2019), DicomViewer from Philips (Philips, 2020) and MITK software from dkfz (GCRF, 2020), Germany for the viewing the images of the patient. These software provides basic features like windowing, MPR visualization, and surface rendering techniques.

*E.2. Classification*

After finalizing the total number of datasets, based on essential parameters, a homogeneous group of datasets is created, which is called classification based on attributes. To test the developed image processing methods during empirical testing, it becomes easy to select the samples based on the parameters of interest. An excel sheet of datasets and the image acquisition parameters are created to refer to the samples (Table 4) quickly. For example, to pick the dataset which is acquired at specific kVp value, directly, kVp column is selected in this excel sheet. This filters the datasets acquired at specific kVp. This approach of selecting the required parameters of interest in the excel sheet reduces the time for searching the entire database. 

*F. Data Validation*

**Figure 4:**
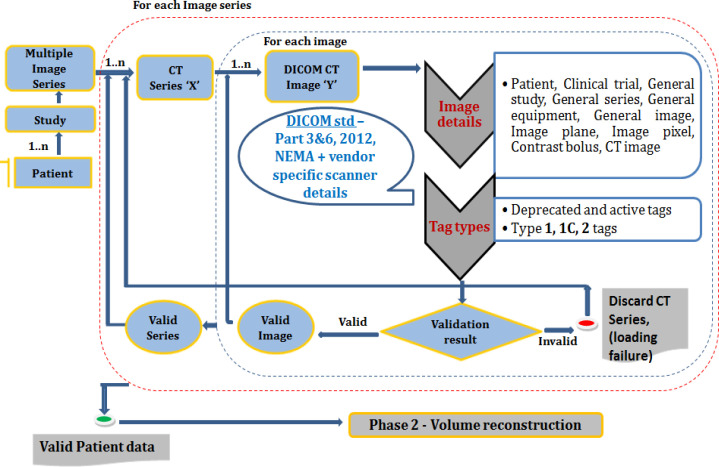
The Design of the DICOM CT Image Validation Framework. The dataset is mainly checked for type 1 and type 2 attributes as per the DICOM standards (DICOM).

DICOM image validation is a prerequisite step in any medical image processing research before using the dataset for empirical testing to check the completeness and uniqueness of the header details. Even though the CTC images from NCI have passed the data completeness verification (Smith et al., 2016), to safeguard from using incomplete data according to the latest standard (NEMA 2020, Philips, 2018; Philips, 2013; Siemens, 2012), a DICOM validation framework is implemented (Figure 4). Dataset is validated for type 1 and type 2 attributes. Type 3 validations were performed only for few tags as their values are not significant. Upon selection of the CT image series, the files are opened and read for the DICOM data elements using parallel processing. The slice location tag has its value stored in two different tags. Generally, if the value is not available in the tag (0020, 1041), then it has to be considered from the z component of the Image position tag (0020, 0032). After reading all images, they are sorted in the ascending order of slice location (in z direction) to check the missing slices. Then, across CT images, specific modules (Table 5) are verified for the uniqueness of the values. For any missing tags, missing slices, and if specific tag values are not unique, the datasets are discarded. 158 out of 164 samples have passed validation. This framework can be generalized for other modalities like MRI, PET, and US (ultrasound) also.

Some of the manufacturer’s specific private tags (Philips, 2018; Philips, 2013; Siemens, 2012) are also considered as defined in the DICOM conformance statement, which was part of the DICOM standard 2015 prior release. By considering the private tags, the backward compatibility of different versions is achieved. Old datasets might not work if the tags are ignored. Clinical trial and contrast bolus modules are not considered for validation as they were removed when data was anonymized. Seven datasets were failed during DICOM validation, as type 2 attributes were empty without any values.

## Results

In the tabulated data (table 4), the number of dataset instances obtained based on the parameter of interest are,

• Image quality: Good diagnostic quality – 166 datasets, bad diagnostic quality – 21 datasets

• Milliampere: 240mA – 61, 200mA – 108, 140mA – 4

• kVp: 120kVp – 185, 100kVP -2

• Age group: 61-70 years: 43, 51 – 60: 80, 41 – 50: 56

• Pixel size in mm: 0.5 – 0.6: 23, 0.6 – 0.7: 68, 0.7 – 0.8: 78, 0.8 – 0.9: 17

• Slice Thickness in mm: 2.5mm – 130, 1mm - 57

**Figure. 5:**
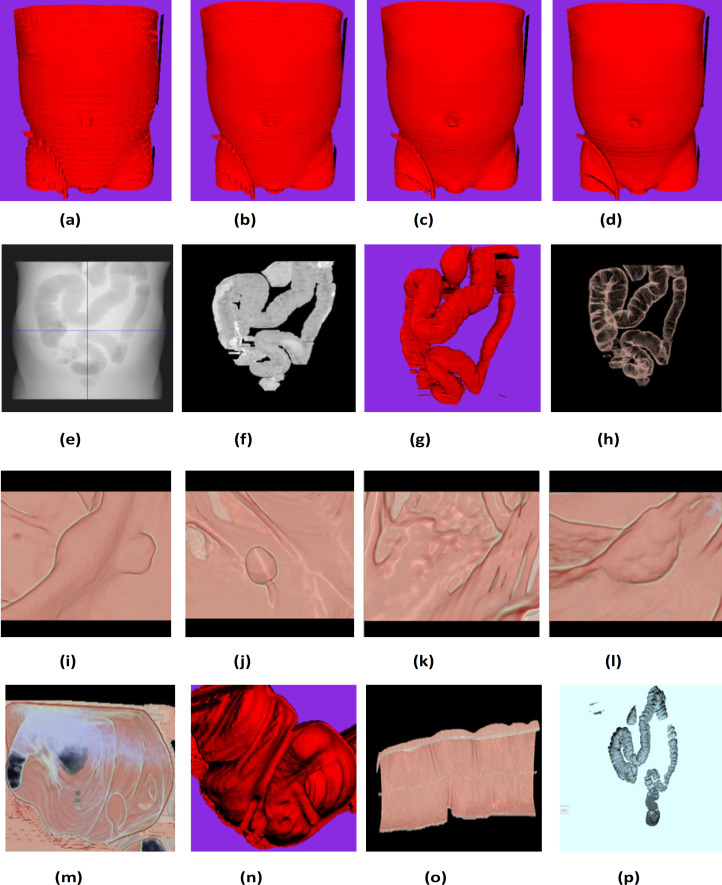
The Result of 3D Volume Reconstruction, Colon Segmentation, and the Endoluminal View. (a) Surface rendering with ST=5.0 mm, (b) 3.0 mm, (c) 1.5 mm (d) 0.75 mm, (e) The DRR image of unsegmented colon, (f) The DRR image of segmented colon, (g) Surface rendering, (h) Direct volume rendering (i-l) The endoluminal view of colon interior showing the colonic structures, (m) The cloudy appearance of the carbon-di-oxide used for insufflating the colon for distension, (n) The splenic flexure, (o) The cross-section of the ascending colon in 3D view and (p) The non-distended colon identified after colon segmentation

After dataset validation as per the DICOM standard, the 3D volume is reconstructed, segmented the VOI (colon), and measured the smaller polyps. Exploratory research in polyp analysis is the potential application of this dataset and also for the clinical validation. Datasets have pixel sizes in both x and y axis in the range of 0.546875-0.9765625 mm and in the z axis in the range of 2.5-5.0mm. Since the images have unequal size only in z axis, reconstruction of the 3D volume is done with a linear interpolation technique. Figure 5a-d shows the surface rendering of the raw 3D volume at variable ST. Higher ST (Figure 5a) produces a staircase effect which does not show a smooth transition of anatomies when compared with least ST (Figure 5d). Few studies (Song et al., 2014; Summers, 2010) have reported that polyps are underestimated by ~2mm in CTC due to the absence of isotropic voxels creation. With some dataset, colonic structures were reproduced excellently with the least slice thickness when visualized using direct volume rendering.

**Table 1:** CTC Image Acquisition Parameters from the Downloaded Dataset from NCI (NCI, 2020).

Anatomical site	Abdomen
ST in mm	{ 1.25, 2.5, 5 } mm
kVp (peak kilo voltage)	{ 100, 120 }
Pixel size in mm	{ 0.58 – 0.93 } square size pixels
Radiometric Resolution	16 bit
CT images/position scan	~ 1000 (for both FFS and FFP)
Machine manufacturer	SIEMENS Sensation 16, 64TM, GE Lightspeed 16^TM^, Philips Brilliance 16TM, Toshiba 64TM
Modalities	CT
Dimensions	2D, 3D
mA (milli ampere)	{ 60, 100, 120, 140, 141, 200, 240, 250, 280, 300 }
Image Resolution	512,512 and 1024x1024
Patient positions	{ FFS, FFP, FFS+FFP, HFS+HFP }
Multi Detector CT	8/16 slices

**Table 2:** CT Dataset Search Results Based on the Parameters of Interest, Mainly the Image Acquisition Parameters, the Image Equipment Details and the Study Dates (NCI, 2020, https://ncia.nci.nih.gov/ncia/login.jsf, as of Jan 2016).

Acquisition Matrix	(= 511.0 and <= 512.0)
Collection(s)	Virtual Colonoscopy
Convolution Kernel	7.200000 mm, B, B19f, B20f, B20s,B30f,B30s, B31f, B31s, B40s,B41f,B41s, B45f,B50f,B70f,B70s,B80f,B80s,BONE, C, D, DETAIL, EXXPERIMENTAL 7, FC01,FC02, FC03,FC10,FC11,FC13,FC50, FL01,FL02, H30S, LUNG, PET AC, Rad:, SOFT, STANDARD, T20s, T80s,ua,ub, X-Y-Z Guassian F, XYZ G7 .00, XYZ G7.00, XYZ Guass5.00, XYZ Guass7.00, hanning
Date available on NCIA	12/11/2002 - 12/09/2013
Image Modality	CT
"Image Slice Thickness (non-ultrasound"	(>0.0 and <=2.0)
Kilovoltage Peak Distribution	(>80.0 and <120.0)
Manufacturer	Philiips Medical Systems, GE, CMS, Inc., CPS, KODAK, General Electric, VARIAAN Medical Systems, Swissray, Philips Medical Systems Inc, Radiology Research, TOSHIBA, AGFA, GEMS, \"GE HEALTHCARE\", FUJI PHOTO FILM Co., ltd., Agfa-Gevaert AG, N/A. Siemens, GE MEDICAL SYSTEMS, SIEMENS, PowerDicom, DeJamette Research Systems, Normalized to NAWM Cr, Philips, Multiple
NBIA Nodes Return cases that include	"http:// imaging.nci.nih.gov:80/wsrf/services/cagrid/NCIACoreService, http://niams-imaging.nci.nih.gov:80/wsrf/services/cagrid/NCIACoreService" any of these modalities

**Table 3:** Reasons for Discarding Dataset from Study (ACR, 2020; Johnson, 2016)

Sl No.	Reasons for bad diagnostic quality
1	Inadequate distension (Figure 3b)
2	CT incomplete - the patient could not retain air (Figure 3a)
3	Debris in sigmoid and splenic flexure, loss of air and retained stool
4	Diverticulosis – non-distended
5	Patient too large (Figure 3b)
6	Streak artifact from right hip arthroplasty (Figure 3c)

**Table 4:** Tabulation of Samples (Based on Attributes) for Easy Access during Hypothesis testing. The DICOM validation column shows the validation status of the dataset

SubjectID	Patient ID	"Image quality"	DICOM validation	Metal artifact	Polyp found	"Slice thickness"	kVp	mA	Contrast	pixel spacing	"Filter Kernel"	Image dimension	W, L	Patient position	Slices	Colon segment	Age	Gender	Manufacturer	Description	Study Date
SD VC 003M	1.3.6.1.4.1.9328.50.6.554	Good			No	2.5	120	240		0.703125	SOFT	512,512	400,40	FFS,FFP	434,453		40	M			2000
SD VC 006M	1.3.6.1.4.1.9328.50.6.3294	Good			Yes	2.5	120	240		0.730469	SOFT	512,512	400,40	FFS,FFP	437,436		60	M			2000
SD VC 008M	1.3.6.1.4.1.9328.50.6.4247	Good			Yes	2.5	120	240		0.681641	SOFT	512,512	400,40	FFS,FFP	437,424		50	M			2000
SD VC-197M	1.3.6.1.4.1.9328.50.6.103932	Bad			Yes	2.5	120	200		0.650391	SOFT	512,512	400,40	FFS,FFP	356,378		50	F			2000
SD VC-396M	1.3.6.1.4.1.9328.50.6.230471	Good			Yes	2.5	120	200		0.734375	SOFT	512,512	400,40	FFS,FFP	426,438		60	M			2000
SD VC-397M	1.3.6.1.4.1.9328.50.6.231340	Good			Yes	1.25	120	200		0.796875	SOFT	512,512	400,40	FFP	993		70	M			2000
SD VC-401M	1.3.6.1.4.1.9328.50.6.234940	Good			Yes	2.5	120	200		0.703125	SOFT	512,512	400,40	FFS,FFP	422,9		60	M			2000
WRAMC VC-001M	1.3.6.1.4.1.9328.50.99.1	Good			Yes	1.25	120	200		0.625	STANDARD	512,512	400,40	FFS,FFP	413,451		50	M			2000
WRAMC VC-080M	1.3.6.1.4.1.9328.50.99.63574	Good			Yes	1.25	120	200		0.664062	STANDARD	512,512	400,40	FFS,FFP	438,473		50	M			2000
WRAMC VC-091M	1.3.6.1.4.1.9328.50.99.79133	Good			Yes	1.25	120	200	<Contrast	0.78125	STANDARD	512,512	400,40	FFS,FFP	399,408		70	M			2000
WRAMC VC-092M	1.3.6.1.4.1.9328.50.99.80603	Good			Yes	1.25	120	200		0.78125	STANDARD	512,512	400,40	FFS,FFP	448,485		50	M			2000
WRAMC VC-100M	1.3.6.1.4.1.9328.50.99.75748	Good			Yes	1.25	120	200		0.664062	STANDARD	512,512	400,40	FFS,FFP	414,423		60	M			2000
SD VC-394M	1.3.6.1.4.1.9328.50.6.228684	Bad			Yes	2.5	120	200		0.689453	SOFT	512,512	400,40	FFS	414		60	F			2000
WRAMC VC-023M	1.3.6.1.4.1.9328.50.99.19129	Bad				1.25	120	200		0.683594	STANDARD	512,512	400,40	FFS,FFP	440,451		60	M			2000
WRAMC VC-032M	1.3.6.1.4.1.9328.50.99.40671	Bad			Yes	1.25	120	200		0.644531	STANDARD	512,512	400,40	FFS,FFP	387,404		70	F			2000
WRAMC VC-050M	1.3.6.1.4.1.9328.50.99.24127	Bad				1.25	120	200		0.625	STANDARD	512,512	400,40	FFS,FFP	403,422		50	F			2000
WRAMC VC-116M	1.3.6.1.4.1.9328.50.99.90702	Bad				1.25	120	200		0.625	STANDARD	512,512	400,40	FFS,FFP	410,390		60	M			2000
WRAMC VC-117M	1.3.6.1.4.1.9328.50.99.88110	Bad				1.25	120	200		0.839844	STANDARD	512,512	400,40	FFS,FFP	412,464		50	M			2000

**Table 5:** List of DICOM CT Modules Validated (as per DICOM PS 3.3, 2020b) (NEMA, 2020)

Sl. No	Module	Tag types	Validated	Defined in page
1	Patient	2	Yes	C.7.1.1, pp. 446
2	General study	2	Yes	C.7.2.1, pp. 488
3	General Series	1, 2	Yes	C.7.3.1, pp. 495
4	General Equipment	1C	Yes	C.7.5.1, pp. 508
5	General image	2	Yes	C.7.6.1, pp. 513
6	Image plane	1, 2, 3	Yes	C.7.6.2, pp. 519
7	CT Image	1, 2	Yes	C.8.2.1, pp. 620
8	Image pixel	1, 2, 1C	Yes	C.7.6.3, pp. 521
9	Clinical trial	1, 2, 1C	No	C.34.4.1, pp. 1562
10	Contrast bolus	2, 3	No	C.7.6.4, pp. 532
11	CT acquisition	3	Yes	A.81.4, pp. 418

**Table 6:** Freely Downloadable Radiology Images from Other Universities and Radiology Centers

Sl. No	Image Source	URL reference
1	Cancer Imaging Archive	https://www.cancerimagingarchive.net/
2	CT Medical Images	https://www.kaggle.com/kmader/siim-medical-images
3	NCBI – Medical Image Databases	https://www.ncbi.nlm.nih.gov/pmc/articles/PMC61234/
4	NIH Database of 100,000 Chest X-Rays	https://nihcc.app.box.com/v/ChestXray-NIHCC
5	Open-Access Medical Image Repositories	http://www.aylward.org/notes/open-access-medical-image-repositories
6	The Berkeley Segmentation Dataset and Benchmark	https://www2.eecs.berkeley.edu/Research/Projects/CS/vision/bsds/
7	Online Medical Images	http://www.onlinemedicalimages.com/index.php/en/
8	UCL – Medical Image Repositories	https://www.ucl.ac.uk/child-health/support-services/library/resources-z/medical-image-repositories
9	DERMOFIT Image Library	http://licensing.eri.ed.ac.uk/i/software/dermofit-image-library.html

## Discussion

Segmenting the VOI from the 3D volumetric data is an important step before polyp analysis. A new boundary-based semi-automatic colon segmentation (Figure 5e-h) method was developed, which works on the knowledge of colon distension grading (Manjunath et al., 2016). Figure 5 shows the results of segmentation. Figure 5e, and Figure 5f shows the colon distribution on DRR (artificial X-Ray) image before and after segmentation, respectively. The results and unsegmented volume are compared through DRR images. Figure 5g-5h illustrates the surface rendered (with marching cube algorithm (Bourke, 2013)) and direct volume rendered (with Microsoft Volume Rendering Framework (Melancon et al., 2016)) images. Figure 5i-5l shows the endoluminal view of the colon interior, and a few cases are a smaller polyp (Figure 5i), a pedunculated polyp (Fig. 5j), floating fecal matter (Figure 5k) and a polyp on the haustral fold (Figure 5l).

The implementation of the work includes Microsoft.NET Framework 4.7.2 and C# programming language with object-oriented design and multithread programming for parallel processing. The system workstation configuration is Intel Xeon^®^ CPU E52620 2.0GHz, 64GB DDR3 RAM, NVidia 4GB GPU, Microsoft Visual Studio 2017, Microsoft .NET Framework 4.7.2, and Accord .NET Framework 2.8.2 (Souza, 2016). At present in addition to CTC dataset, we also have the dataset of different cancers such as Glioblastoma Multiforme (CPTAC-GBM), Cutaneous Melanoma (CPTAC-CM), Non-small Cell Lung Cancer (NSCLC-Radiomics-Interobserver1, NSCLC-Cetuximab, and QIN LUNG CT), Ductal Adenocarcinoma (CPTAC-PDA), Head and Neck Cancer (Head-Neck-Radiomics-HN1), Clear Cell Carcinoma (TCGA-KIRC), Bladder Endothelial Carcinoma (TCGA-BLCA) and Corpus Endometrial Carcinoma (CPTAC-UCEC) and working on feature-based machine learning techniques. 

*Limitations of the study*

Despite the vast dataset, the TCIA collection has limited samples of the CTC images acquired at different levels of kVp and with least slice thickness such as 0.625mm, 0.5 mm etc.. There were no images in the collection apart from 120kVP and 100kVp. Empirical testing of virtual colon cleansing required the images acquired with different kVp values. There are many datasets where the patient’s body lies outside the scan field of view. It is a time-consuming task to process such images. Other image database from different University hospitals and government supported research centers are available freely for the research community. Few of these are shown in Table 6.

NCI dataset is a source of inspiration for any researcher working in medical image processing. With this dataset, automated methods have been developed for DICOM data validation, colon segmentation, Electronic Cleansing, and smaller polyp measurement. As the dataset collection is too vast, the researcher should be careful in sample design and collection on which the statistical analysis of the results completely depends. Therefore it is essential to classify the dataset based on the attributes of interest and to prepare an index sheet that simplifies the empirical testing based on the parameters of interest. This approach even helps in continuing with machine learning of medical big data images. Further, the scope of the work is on other anatomical sites and other cancer types to develop decision-making systems and also on the brain tumor quantification using MRI dataset from TCIA-Glioblastoma collection. This study successfully researched the TCIA CT Colonography collection. It is good if the datasets with the least slice thickness images are also available.

## Data Availability

The datasets analysed during the current study are available in the National Cancer Institute repository, https://public.cancerimagingarchive.net/ncia/login.jsf (http://doi.org/10.7937/K9/TCIA.2015.NWTESAY1)

## Summary points

Data collection in medical image analysis is known for the most of the researchers. But collecting them in a systematic way which will simply their empirical testing was not discussed in the papers.
